# Development and Multicentric Validation of a Lateral Flow Immunoassay for Rapid Detection of MCR-1-Producing *Enterobacteriaceae*

**DOI:** 10.1128/JCM.01454-18

**Published:** 2019-04-26

**Authors:** Hervé Volland, Laurent Dortet, Sandrine Bernabeu, Hervé Boutal, Marisa Haenni, Jean-Yves Madec, Frédéric Robin, Racha Beyrouthy, Thierry Naas, Stéphanie Simon

**Affiliations:** a Service de Pharmacologie et Immuno-analyse (SPI), JOLIOT, CEA, INRA, Université Paris-Saclay, Gif sur Yvette, France; b EA7361, UPSUD, Laboratoire de Bactériologie & CNR de la Résistance aux Antibiotiques, Hôpital de Bicêtre, APHP, Le Kremlin Bicêtre, France; c Unité Antibiorésistance et Virulence Bactériennes (AVB), Université de Lyon—Anses, Lyon, France; d CHU Clermont-Ferrand, Laboratoire de Bactériologie & CNR de la Résistance aux Antibiotiques, Clermont-Ferrand, France; Memorial Sloan Kettering Cancer Center

**Keywords:** MCR-1, detection, lateral flow immunoassay

## Abstract

Colistin has become a last-resort antibiotic for the treatment of infections caused by highly drug-resistant Gram-negative bacteria. Moreover, it has been widely used in the livestock sector. As a consequence, colistin resistance is emerging worldwide. Among the colistin resistance mechanisms, the spread of the plasmid-encoded colistin resistance gene *mcr-1* (mostly in Escherichia coli) is of particular concern due to its increased transferability compared to that of chromosome-encoded resistance. The early detection of MCR-1-producing bacteria is essential to prevent further spread and provide appropriate antimicrobial therapy. Lateral flow immunoassays (LFIAs) were manufactured with selected monoclonal antibodies. A collection of 177 human and 121 animal enterobacterial isolates was tested in a multicentric study. One bacterial colony grown on agar plates was suspended in extraction buffer and dispensed on the cassette. Migration was allowed for 15 min, and the results were monitored by the appearance of a specific band. The positive results showed a pink line resulting in an unambiguous interpretation. All MCR-1-producing isolates were found to be positive by the LFIA, and no false-negative results were observed. Three out of four MCR-2-producing isolates were also found to be positive. Our test does not detect MCR-3-, MCR-4-, or MCR-5-producing isolates. LFIA allows the detection of MCR-1 with 100% sensitivity and 98% specificity. This test is fast, sensitive, specific, easy to use, and cost-effective and can therefore be implemented in any microbiology laboratory worldwide. LFIA is a major tool for the rapid detection and monitoring of MCR-1 producers in humans and animals.

## Abstract (precis)

Colistin has become a last-resort antibiotic for the treatment of infections caused by highly drug-resistant Gram-negative bacteria. Moreover, it has been widely used in the livestock sector.

## INTRODUCTION

The rise of antimicrobial resistance in association with an extremely limited number of novel molecules with antimicrobial activity is a real threat to global health. This is of particular concern with the worldwide dissemination of multidrug-resistant (MDR) and highly drug-resistant (XDR) Gram-negative bacteria, especially carbapenemase-producing *Enterobacteriaceae* (CPE). The paucity of therapeutic options still available for these MDR and XDR bacteria has led to the revival of polymyxins (polymyxin B and colistin), which have become the last-resort therapy (1). Unfortunately, the increasing prevalence of CPE worldwide has boosted the use of colistin, inexorably resulting in the rise of polymyxin resistance, especially in countries where CPE is endemic, such as Italy and Greece (2–6).

In enterobacterial isolates, acquired resistance to colistin is mostly caused by modifications of the lipopolysaccharide (LPS) (7, 8). In late 2015, the first plasmid-encoded gene causing resistance to polymyxin, named *mcr-1*, was described (9). MCR-1 is a phosphoethanolamine transferase able to add pETN to the LPS, which leads to polymyxin resistance, with MICs usually ranging from 2 to 8 mg/liter. Since its initial description, the *mcr-1* gene has been reported worldwide in *Enterobacteriaceae* (mostly Escherichia coli) recovered from both human and animal samples (7, 10). On top of its ability to be transferred between enterobacterial species, recent reports indicate its very low (or a lack of a) fitness cost for the bacteria, raising fears of the rapid dissemination of this mechanism (11, 12). Until now, eight families of *mcr* genes have already been assigned, and seven have been reported in *Enterobacteriaceae* (13–18). MCR-2, MCR-3, MCR-4, MCR-5, MCR-6, MCR-7, and MCR-8 share only 81%, 34%, 33%, 31%, 82%, 29%, and 31% amino acid sequence identity with MCR-1, respectively. It is of note that MCR-1 remains the most prevalent enzyme in *Enterobacteriaceae* isolated from human samples (19). To contain the spread of MCR-producing *Enterobacteriaceae* in humans, a test that enables both the rapid and reliable detection of at least MCR-1 is urgently needed.

Broth microdilution (BMD) has recently been chosen as the unique reference method by CLSI and by EUCAST (20), even though diffusion methods are still of use in the animal sector (21). Currently, discrimination between chromosome-encoded and MCR-related resistance to colistin mostly relies on molecular assays dedicated to the detection of the *mcr-1* and *mcr-2* genes. These methods are based on real-time PCR (22–24), loop-mediated isothermal amplification (25), and microarray techniques (26). Multiplex PCRs have also been developed for the detection of the five most prevalent families of *mcr* variants (*mcr-1*, *mcr-2*, *mcr-3*, *mcr-4*, and *mcr-5*) (27–29). These molecular techniques remain expensive and sometimes require experienced staff. More recently, a matrix-assisted laser desorption ionization–time of flight mass spectrometry-based assay has been developed for the detection of polymyxin resistance and discrimination between chromosome-encoded and plasmid-encoded resistance to colistin in E. coli (30). However, this promising technique has to be validated in other bacterial species and requires a mass spectrometer, which is not available in all clinical microbiology laboratories. Finally, a simple phenotypic method, named the Colistin-MAC test, has been described for the screening of MCR-1-mediated polymyxin resistance (31). This method is based on the colistin MIC reduction in the presence of dipicolinic acid, when the colistin resistance is caused by MCR-1 production. This method is inexpensive and easy to perform but requires determination of additional MICs using the broth microdilution method, thus leading to an additional delay of at least 24 h.

In this study, we developed a lateral flow immunoassay (LFIA) for the rapid detection (<15 min) of MCR-1 in *Enterobacteriaceae*. We have validated it on a collection of 298 characterized isolates recovered from human and animal samples.

## MATERIALS AND METHODS

### Ethics statement.

All experiments were performed in compliance with French and European regulations on the care of laboratory animals (European Community [EC] Directive 86/609, French Law 2001-486, 6 June 2001), with agreement no. 91-416, delivered to S. Simon by the French Veterinary Services, and with CEA agreement D-91-272-106 from the Veterinary Inspection Department of Essonne, France.

### Reagents.

Biozzi mice were bred at the animal care unit of CEA (Gif sur Yvette, France). Bovine serum albumin (BSA), Tween 20, isopropyl-β-d-thiogalactoside (IPTG), biotin *N*-hydroxysuccinimide ester, streptavidin, gold chloride solution, *N*-succinimidyl-*S*-acetyl-thioacetate (SATA), imidazole, and kanamycin (from Streptomyces kanamyceticus) were from Sigma-Aldrich (Saint Quentin Fallavier, France). The NdeI and XhoI restriction enzymes were from New England Biolabs (Evry, France). Goat anti-mouse (GAM) IgG and IgM polyclonal antibodies were from Jackson ImmunoResearch (West Grove, PA, USA). Protein A Sepharose was from Millipore (ProsepA; Guyancourt, France). A serine protease inhibitor [4-(2-aminoethyl)benzenesulfonyl fluoride hydrochloride (AEBSF)] was from Interchim (Montluçon, France). Metal agarose affinity resin (chelating Sepharose FastFlow) was from GE Healthcare (Vélizy-Villacoublay, France). Enzyme immunoassays (EIAs) were performed with MaxiSorp 96-well microtiter plates (Nunc, Paris, France), and all reagents were diluted in EIA buffer (0.1 M phosphate buffer, pH 7.4, containing 0.15 M NaCl, 0.1% bovine serum albumin [BSA], and 0.01% sodium azide). Plates coated with proteins were saturated in EIA buffer (18 h at 4°C) and washed with washing buffer (0.1 M potassium phosphate, pH 7.4, containing 0.05% Tween 20). Nitrocellulose strips with polystyrene backing were from GE Healthcare (Prima 40). Lysogeny broth (LB) agar plates were from Sigma-Aldrich, and Mueller-Hinton agar plates were from bioMérieux (La Balme-Les-Grottes, France).

### Bacterial isolates.

For the LFIA validation, 298 enterobacterial isolates with a characterized *mcr* content were used to evaluate the *mcr-1* LFIA (see Table S1 in the supplemental material). This collection included 110 MCR-1 producers (1 of which was colistin sensitive), 4 MCR-2 producers, 18 MCR-3 producers (2 of which were colistin sensitive), 5 MCR-4 producers, 3 MCR-5 producers, and 158 non-MCR producers, including 91 colistin-resistant isolates. These isolates were collected and tested at three different locations: Bicêtre Hospital and Clermont-Ferrand Hospital for human samples and Anses Lyon for animal samples.

### Colistin susceptibility testing.

MICs were determined by BMD according to the guidelines of a CLSI and EUCAST joint subcommittee (20). Results were interpreted using the EUCAST breakpoint as updated in 2018 (32), where susceptible was an MIC of ≤2 μg/ml and resistant was an MIC of >2 μg/ml.

### Cloning and expression of the MCR-1_179-541_ protein in E. coli.

A partial region of the *mcr-1* gene encoding the periplasmic region of the protein (from amino acids T179 to R541 [MCR-1_179–541_]) was amplified by PCR using the forward primer MCR-1_179–541_ NdeI (5′-aaaaaaCATATGtatgccagtttctttcgcgtgcat-3′; capital letters correspond to the restriction site) and the reverse primer MCR-1 XhoI-Δstop (5′-aaaaaaCTCGAGgcggatgaatgcggtgcggtcttt-3′). After amplification, the sequence was further cloned into the pET41b vector (Invitrogen Life Technologies, Cergy-Pontoise, France), using the NdeI and XhoI restriction enzymes, allowing insertion of a polyhistidine tag sequence at the 3′ end of the protein. The inserted amplicon was verified by sequencing (Eurofins Genomics, Ebersberg, Germany).

E. coli BL21(DE3)pLysS was then transformed with the recombinant plasmid. One positive clone was grown in 500 ml of LB with 100 µg/ml ampicillin at 37°C until the optical density at 600 nm reached 0.6. IPTG (100 µM) was added to the culture, which was incubated for 4 h with shaking at 37°C. The culture was pelleted by centrifugation at 2,500 × g for 20 min at 4°C and suspended in 30 ml of solubilizing buffer (50 mM Tris-HCl buffer, pH 8, 500 mM NaCl, 1 mM AEBSF, 8 M urea). After solubilization, the bacterial suspension was sonicated (5 pulses of 15 s each at 14 W), incubated at 37°C for 1 h, and centrifuged for 15 min at 20,000 × g. The supernatant was stored at −20°C. Imidazole (final concentration, 20 mM) was added to the supernatant, which was incubated for 1 h with 1 ml of Ni-nitrilotriacetic acid agarose affinity resin with shaking at 4°C. The gel was washed with 25 ml of binding buffer (50 mM Tris-HCl buffer, pH 8, 100 mM NaCl, 8 M urea). Elution of the His-tagged protein was performed by incubating the resin for 10 min with 2 ml of solubilizing buffer with 500 mM imidazole (final concentration), and the operation was repeated 4 times.

The eluted fractions were pooled and dialyzed twice in 2 liters of 50 mM potassium phosphate buffer, pH 7.4. The protein concentration was measured by determination of the absorbance at 280 nm, and purity was assessed by SDS-PAGE (Phast system; GE Healthcare). The purified recombinant periplasmic region of MCR-1 (p-MCR-1) was then used to immunize mice, as a standard for the selection of monoclonal antibody (MAb) pairs, and to determine the limit of detection.

### MCR-1 LFIA evaluation.

The MCR-1 tests (strip and cassette) were manufactured by NG Biotech (Guipry, France) using our MAbs. The 298 strains to be tested were grown on Mueller-Hinton agar (bioMérieux, La Balme-Les-Grottes, France). Using a 1-µl inoculation loop, a single colony was resuspended in 100 µl of extraction buffer and then subsequently dispensed on the cassette for migration. After a 15-min migration, the results were read by eye by monitoring the appearance of a red band specific to MCR-1, along with a band corresponding to the internal control.

### Limit of detection (LOD) with recombinant MCR-1 and MCR-1-producing enterobacterial isolates.

Dilutions (5, 1, 0.5, 0.3, and 0 ng/ml) of the recombinant p-MCR-1 protein or of one MCR-1-producing E. coli strain (6 × 10^8^, 2 × 10^8^, 0.67 × 10^8^, and 0 CFU/ml) were performed in extraction buffer or LB medium, respectively. Bacterial dilution vials were centrifuged for 20 min at 6,000 × *g* at 4°C, the supernatant was carefully discarded, and the pellet was suspended in extraction buffer (at the same volume as LB). One hundred microliters of each solution was consecutively dispensed on the cassette and allowed to migrate. The results were read by eye after 15 min.

### Routine use of the manufactured LFIA.

Five drops of an extraction buffer were delivered into a vial (both of which were provided in the kit). A colony was collected from a culture plate with an inoculation loop (not provided in the kit). The colony was resuspended in the extraction buffer by vigorous stirring. A lid was placed on the vial, and the vial was briefly vortexed for a few seconds (lysis step). One hundred microliters of the extract was consecutively directly delivered onto the cassette with a calibrated disposal pipette (provided in the kit). Migration was allowed for 15 min before the results were read by eye.

## RESULTS

### Combinatorial test and best pair selection.

Recombinant p-MCR-1 (1.3 mg) was obtained from 500 ml of culture. SDS-PAGE (see Fig. S1 in the supplemental material) under nonreducing conditions and reducing conditions showed a major band with a molecular mass corresponding to the theoretical expected molecular mass of 40 kDa. This protein was then used to immunize mice. Twenty MAbs (named 101 to 120) were finally selected. Four hundred pairs were tested during the first part of a combinatorial study done with spotted strips and recombinant p-MCR-1. During the second part of the combinatorial study, 48 pairs were tested with extracted MCR-1. The 3 pairs of antibodies displaying the strongest specific signal with a specific U-shaped signal revealing a high-affinity capture antibody (33) and no nonspecific signals were further selected (MCR1-108/MCR1-110*, MCR1-114/MCR1-110*, and MCR1-116/MCR1-110*, where * indicates the colloidal gold conjugate antibody). In order to discriminate the best pair among these three pairs, they were further tested with serial dilutions of MCR-1-expressing strains. The MCR1-116/MCR1-110 pair, which showed the lowest limit of detection was selected, and a batch of 1,000 tests (strip plus cassette) was produced (NG Biotech) in order to carry out the validation assay (sensitivity, specificity).

### LOD using the MCR-1 LFIA.

**FIG 1:**
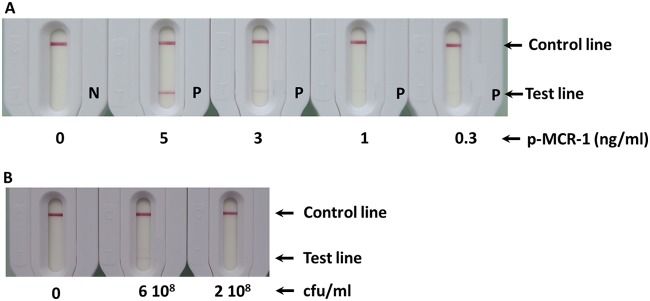
Limit of detection in extraction buffer. (A) Serial dilutions with recombinant p-MCR-1. P, positive; N, negative. (B) Serial dilutions with MCR-1-expressing Escherichia coli.

Although the positive test lines at 300 pg/ml and 2 × 10^8^ CFU/ml are not visible in Fig. 1 (due to the camera sensitivity), these lines were clearly seen visually. Thus, a limit of detection (LOD) of about 300 pg/ml for recombinant protein and 2 × 10^8^ CFU/ml for an MCR-1-producing E. coli strain was determined by eye after 15 min of migration (Fig. 1).

**FIG 2:**
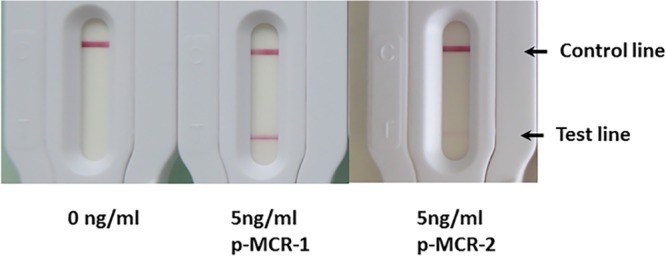
Comparison of detection of recombinant p-MCR-1 and p-MCR-2.

We also compared the results obtained with p-MCR-1 and p-MCR-2 (produced using the same protocol used for p-MCR-1). Figure 2 shows that we obtained signals for the same protein concentration of p-MCR-2. This is probably due to the cross-reactivity of our antibodies with this protein.

### Performance of the MCR-1 LFIA with reference isolates.

As described in Table 1, the MCR-1 LFIA was able to detect all 109 colistin-resistant MCR-1-producing isolates whatever the location: Bicêtre Hospital, Clermont-Ferrand Hospital, or Anses Lyon. One *mcr-1*-positive E. coli isolate that remained colistin susceptible (MIC, below 0.5 mg/liter) gave a negative result, suggesting that the *mcr-1* gene either was not expressed or was expressed at a low level that was below the limit of detection of the test and that did not allow the bacteria to be resistant to colistin. Three out of 4 MCR-2-producing strains gave a positive result. All the strains producing MCR-3, -4, and -5 (25 strains) gave negative results. All the non-MCR-producing strains (colistin resistant or susceptible) gave negative results. The result obtained with different isolates are shown in Fig. S2. This validation experiment showed that our test was able to detect colistin-resistant MCR-1-producing *Enterobacteriaceae* with 100% sensitivity and 98% specificity (detection of 3 out of 4 MCR-2-producing bacteria).

**TABLE 1:** Results of MCR-1 LFIA and colistin MICs for a collection of strains comprising MCR and non-MCR producers

Mechanism of polymyxin resistance	Bacterial species	No. of isolates	Colistin MIC (μg/ml)	MCR-1 IFIA result^*a*^
*mcr-1*	E. coli	94	≥2	P
*mcr-1*	Klebsiella pneumoniae	6	≥4	P
*mcr-1*	*Salmonella*	6	≥4	P
*mcr-1*	Salmonella enterica serovar *Typhimurium*	1	8	P
*mcr-1.5*	E. coli	2	4	P
*mcr-2*	E. coli	4	4	P^*b*^
*mcr-3*	E. coli	16	≥1	N
*mcr-4*	E. coli	3	4	N
*mcr-5*	E. coli	2	8	N
*mcr-5*	*Salmonella*	1	8	N
Non-MCR production, colistin resistant	E. coli	31	≥4	N
Non-MCR production, colistin resistant	K. pneumoniae	42	≥4	N
Non-MCR production, colistin resistant	Enterobacter cloacae	15	≥8	N
Non-MCR production, colistin resistant	Citrobacter freundii	1	16	N
Non-MCR production, colistin resistant	Hafnia alvei	2	≥4	N
*mcr-1* ESBL^*c*^, colistin susceptible	E. coli	1	<0.5	N
*mcr-3* ESBL, colistin susceptible	E. coli	2	<0.5	N
*mcr-4* ESBL, colistin susceptible	Shewanella oneidensis	1	0.5	N
*mcr-4* ESBL, colistin susceptible	Shewanella profunda	1	0.5	N
Non-MCR production, colistin resistant	E. coli	66	<0.5	N
Non-MCR production, colistin resistant	E. cloacae	1	0.5	N

a P, positive result; N, negative result.

b Three of four isolates were positive.

c ESBL, extended-spectrum beta-lactamase.

## DISCUSSION

In order to obtain monoclonal antibodies directed against MCR-1, we initially made several attempts to produce the whole protein. All these attempts failed because this protein systematically precipitated after purification. We therefore decided to produce the periplasmic soluble region of this protein (34), which preserves the enzymatic activity of MCR-1, even with a polyhistidine tag (35). The latter observation led us to think that if the periplasmic region kept the enzymatic activity, it should also keep a conformation close to that of whole MCR-1. Using this strategy, we successfully obtained antibodies able to recognize full native MCR-1 by immunizing mice with the periplasmic region (see Materials and Methods S1 in the supplemental material).

All the screening steps until the selection of 20 MAbs were performed using an immunoenzymatic assay to test the capacity of the MAbs to bind recombinant p-MCR-1. The assay conditions of this screening format are very different from those of LFIA (in terms of the kinetics of binding, the reagents, and the assay procedure used) and are not fully predictive of antibody performance in LFIAs (33). In order to select the most appropriate MAbs for the LFIA format, we performed a combinatorial analysis using operating conditions close to those of the LFIA format (see Materials and Methods S2 in the supplemental material). We had previously observed that the best pair for the detection of recombinant protein is not systematically the best for the detection of the natural protein, and the risk was even greater in this study, where we used a truncated recombinant protein. Therefore, the final selection was performed using serial dilutions of MCR-1-producing isolates. This resulted in the selection of a pair of antibodies, MCR1-116 as the capture antibody and MCR1-110 as the colloidal gold reporter antibody, which were used for validation of the test. The LOD of this LFIA was 300 pg/ml for p-MCR-1 and 2 × 10^8^ CFU/ml for MCR-1-producing *Enterobacteriaceae*. The LOD obtained with p-MCR-1 is close to the LODs previously obtained for the 5 main carbapenemases (36). Unfortunately, the results varied greatly from one batch of recombinant protein to another, probably because of the stability of the protein. We therefore evaluated the performance of our test using an extract of an MCR-1-expressing strain, as it is more representative of what we expect to detect in a real sample and it allowed us to have a constant limit of detection from one batch to another. Contrary to the LOD obtained with recombinant proteins, the LOD calculated with the bacterial extract is very different from the LODs previously described for the 5 main carbapenemases, 10^8^ versus 10^6^ CFU/ml. This discrepancy might result from (i) the lower accessibility of this protein anchored to the cytoplasmic membrane compared to the β-lactamases present in the periplasm, (ii) a smaller amount of MCR than β-lactamases per bacterium, (iii) a lower efficiency of extraction from membranes, and (iv) a greater decrease in antibody affinity for native proteins than for the recombinant protein for MCR-1 by comparison to what was observed for β-lactamases.

To date, eight *mcr* gene families have been reported in *Enterobacteriaceae*. The *mcr-1* gene is the most prevalent in bacterial isolates recovered from human samples, while the other *mcr* families seem to be more widespread in animals. Currently, the detection of MCR-producing isolates relies on molecular-based assays, including real-time PCR (22–24), loop-mediated isothermal amplification (25), microarray techniques (26), and in-house multiplex PCRs (27–29). Although these techniques are usually highly sensitive and specific, a positive result does not necessarily imply a phenotypic expression, and this absence of phenotype has recently been described for *mcr-3*- and *mcr-4*-positive isolates (37). Conversely, immunoenzymatic assays detect the protein produced. In this study, one *mcr-1*-positive E. coli isolate was not detected by the LFIA. Since this isolate was found to be fully susceptible to colistin (MIC, <0.5 mg/liter), we might assume that the *mcr-1* gene either was not expressed in this strain or was expressed at a low level that was below the limit of detection of the test and that did not allow the bacteria to be resistant to colistin.

The detection of 3 out of 4 isolates producing MCR-2 was due to the cross-reactivity of our antibodies for this protein. The difference in results obtained with these 4 bacteria producing MCR-2 is probably due to a difference in MCR-2 production per bacterium. Indeed, the negative isolate was found to be positive by increasing the quantity of bacteria collected by extraction of 2 1-µl inoculation loops.

### Conclusion.

Since the LFIA is able to efficiently detect MCR-1 and, sometimes, MCR-2, it seems to be a reliable assay for the rapid detection of MCR producers in clinical microbiology laboratories dedicated to human health. One limitation of this LFIA is its restricted spectrum for the detection of MCR-1 and, potentially, MCR-2 variants. Indeed, since the MCR variants are often found in enterobacterial isolates recovered from animal samples (38–41), addition of the most prevalent variants (at least MCR-3, -4, and -5) would be of great interest with a view to using this test to detect MCR producers among colistin-resistant isolates of animal origin.

## ACKNOWLEDGMENTS

This work was funded by a grant from the Ministère de l’Education Nationale et de la Recherche (EA7361), Université Paris Sud, Université Paris Saclay. We are members of the Laboratory of Excellence LERMIT, supported by a grant from ANR (ANR-10-LABX-33).

## Supplementary Material

Supplemental file 1

Supplemental file 2

Supplemental file 3

Supplemental file 4
