# Supplementary material for: Development and Multicentric Validation of a Lateral Flow Immunoassay for Rapid Detection of MCR-1-Producing Enterobacteriaceae
Source: J Clin Microbiol. 2019 Apr 26;57(5):e01454-18. doi: 10.1128/JCM.01454-18 (PMC6498016; doi:10.1128/JCM.01454-18)
Supplement: Supplemental file 1 [file JCM.01454-18-s0001.pdf]

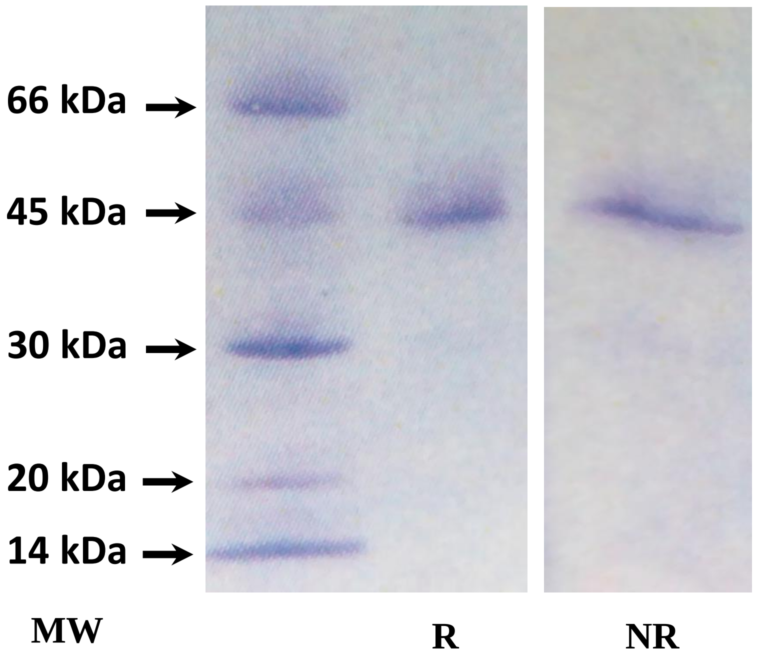

Supplemental Figure 1: SDS-PAGE (Pharmacia Phast system). Purified recombinant MCR-1 (0,34 mg/mL), Coomassie blue staining. MW = molecular weight, NR = non-reducing conditions, R = reducing conditions. (Same gel spliced to avoid the presence of other samples)
