# Supplementary material for: Development and Multicentric Validation of a Lateral Flow Immunoassay for Rapid Detection of MCR-1-Producing Enterobacteriaceae
Source: J Clin Microbiol. 2019 Apr 26;57(5):e01454-18. doi: 10.1128/JCM.01454-18 (PMC6498016; doi:10.1128/JCM.01454-18)
Supplement: Supplemental file 2 [file JCM.01454-18-s0002.pdf]

Supplemental Table 1 . List of enterobacterial isolates susceptible and resistant to colistin of human and animal origin

resistance mechanism to polymyxins	Bacterial species	Colistin MIC	Origin
mcr-1	*E. coli*	4	KB/human
mcr-1	*E. coli*	8	KB/human
mcr-1	*E. coli*	4	KB/human
mcr-1	*E. coli*	4	KB/human
mcr-1	*E. coli*	4	KB/human
mcr-1	*E. coli*	4	KB/human
mcr-1	*E. coli*	4	KB/human
mcr-1	*E. coli*	4	KB/human
mcr-1	*E. coli*	4	KB/human
mcr-1	*E. coli*	4	KB/human
mcr-1	*E. coli*	4	KB/human
mcr-1	*E. coli*	4	KB/human
mcr-1	*E. coli*	4	KB/human
mcr-1	*E. coli*	4	KB/human
mcr-1	*E. coli*	4	KB/human
mcr-1	*E. coli*	4	KB/human
mcr-1	*E. coli*	4	KB/human
mcr-1	*E. coli*	4	KB/human
mcr-1	*E. coli*	4	KB/human
mcr-1	*E. coli*	4	KB/human
mcr-1	*E. coli*	4	KB/human
mcr-1	*E. coli*	8	KB/human
mcr-1	*E. coli*	4	KB/human
mcr-1	*E. coli*	4	KB/human
mcr-1	*E. coli*	4	CF/human
mcr-1	*E. coli*	4	CF/human
mcr-1	*E. coli*	4	CF/human
mcr-1	*E. coli*	4	CF/human
mcr-1	*E. coli*	4	CF/human
mcr-1	*E. coli*	2	CF/human
mcr-1	*E. coli*	8	CF/human
mcr-1	*E. coli*	8	CF/human
mcr-1	*E. coli*	4	CF/human
mcr-1	*E. coli*	4	CF/human
mcr-1	*E. coli*	4	CF/human
mcr-1	*E. coli*	4	CF/human
mcr-1	*E. coli*	4	CF/human
mcr-1	*K. pneumoniae*	32	KB/human
mcr-1	*K. pneumoniae*	4	KB/human
mcr-1	*K. pneumoniae*	4	KB/human
mcr-1	*K. pneumoniae*	32	CF/human
mcr-1	*K. pneumoniae*	16	CF/human
mcr-1	*K. pneumoniae*	8	CF/human
mcr-1	*Salmonella*	4	KB/human
mcr-1	*Salmonella*	8	KB/human
mcr-1	*Salmonella*	8	KB/human
mcr-1	*Salmonella*	8	KB/human
mcr-1	*Salmonella*	8	KB/human
mcr-1	*Salmonella*	8	KB/human
mcr-1	*Salmonella thyphimurium*	8	CF/human
mcr-1	*E. coli*	2	A/animal
mcr-1	*E. coli*	2	A/animal
mcr-1	*E. coli*	4	A/animal
mcr-1	*E. coli*	4	A/animal

mcr-1	*E. coli*	4	A/animal
mcr-1	*E. coli*	8	A/animal
mcr-1	*E. coli*	8	A/animal
mcr-1	*E. coli*	8	A/animal
mcr-1	*E. coli*	8	A/animal
mcr-1	*E. coli*	16	A/animal
mcr-1	*E. coli*	16	A/animal
mcr-1	*E. coli*	16	A/animal
mcr-1	*E. coli*	16	A/animal
mcr-1	*E. coli*	32	A/animal
mcr-1	*E. coli*	64	A/animal
mcr-1	*E. coli*	16	A/animal
mcr-1	*E. coli*	8	A/animal
mcr-1	*E. coli*	4	A/animal
mcr-1	*E. coli*	8	A/animal
mcr-1	*E. coli*	4	A/animal
mcr-1	*E. coli*	2	A/animal
mcr-1	*E. coli*	8	A/animal
mcr-1	*E. coli*	8	A/animal
mcr-1	*E. coli*	8	A/animal
mcr-1	*E. coli*	8	A/animal
mcr-1	*E. coli*	8	A/animal
mcr-1	*E. coli*	8	A/animal
mcr-1	*E. coli*	8	A/animal
mcr-1	*E. coli*	8	A/animal
mcr-1	*E. coli*	8	A/animal
mcr-1	*E. coli*	8	A/animal
mcr-1	*E. coli*	8	A/animal
mcr-1	*E. coli*	8	A/animal
mcr-1	*E. coli*	8	A/animal
mcr-1	*E. coli*	8	A/animal
mcr-1	*E. coli*	8	A/animal
mcr-1	*E. coli*	8	A/animal
mcr-1	*E. coli*	16	A/animal
mcr-1	*E. coli*	16	A/animal
mcr-1	*E. coli*	8	A/animal
mcr-1	*E. coli*	16	A/animal
mcr-1	*E. coli*	8	A/animal
mcr-1	*E. coli*	4	A/animal
mcr-1	*E. coli*	16	A/animal
mcr-1	*E. coli*	4	A/animal
mcr-1	*E. coli*	16	A/animal
mcr-1	*E. coli*	16	A/animal
mcr-1	*E. coli*	8	A/animal
mcr-1	*E. coli*	8	A/animal
mcr-1	*E. coli*	32	A/animal
mcr-1	*E. coli*	16	A/animal
mcr-1	*E. coli*	12	A/animal
mcr-1	*E. coli*	16	A/animal
mcr-1	*E. coli*	6	A/animal
mcr-1	*E. coli*	8	A/animal
mcr-1	*E. coli*	8	A/animal
mcr-1	*E. coli*	16	A/animal
mcr-1.5	*E. coli*	4	KB/human
mcr-1.5	*E. coli*	4	KB/human
mcr-2	*E. coli*	4	KB/human
mcr-2	*E. coli*	4	KB/human
mcr-2	*E. coli*	4	KB/human
mcr-2	*E. coli*	4	CF/human

mcr-3	*E. coli*	4	KB/human
mcr-3	*E. coli*	4	KB/human
mcr-3	*E. coli*	4	CF/human
mcr-3	*E. coli*	4	CF/human
mcr-3	*E. coli*	4	A/animal
mcr-3	*E. coli*	4	A/animal
mcr-3	*E. coli*	2	A/animal
mcr-3	*E. coli*	4	A/animal
mcr-3	*E. coli*	1	A/animal
mcr-3	*E. coli*	4	A/animal
mcr-3	*E. coli*	4	A/animal
mcr-3	*E. coli*	4	A/animal
mcr-3	*E. coli*	4	A/animal
mcr-3	*E. coli*	4	A/animal
mcr-3	*E. coli*	2	A/animal
mcr-3	*E. coli*	4	A/animal
mcr-4	*E. coli*	4	KB/human
mcr-4	*E. coli*	4	KB/human
mcr-4	*E. coli*	4	KB/human
mcr-4	*S. oneidensis*	0.5	KB/human
mcr-4	*S. profunda*	0.5	KB/human
mcr-5	*E. coli*	8	KB/human
mcr-5	*E. coli*	8	KB/human
mcr-5	*Salmonella*	8	KB/human
non-MCR producing strains colistin resistant	*E. coli*	16	KB/human
	E. coli	16	KB/human
	E. coli	4	KB/human
	E. coli	16	KB/human
	E. coli	4	CF/human
	E. coli	4	CF/human
	E. coli	16	CF/human
	E. coli	8	CF/human
	E. coli	8	CF/human
	E. coli	8	CF/human
	E. coli	16	CF/human
	E. coli	4	CF/human
	K. pneumoniae	8	KB/human
	K. pneumoniae	32	KB/human
	K. pneumoniae	16	KB/human
	K. pneumoniae	16	KB/human
	K. pneumoniae	32	KB/human
	K. pneumoniae	32	KB/human
	K. pneumoniae	32	CF/human
	K. pneumoniae	>=64	CF/human
	K. pneumoniae	>=64	CF/human
	K. pneumoniae	>=64	CF/human
	K. pneumoniae	32	CF/human
	K. pneumoniae	8	CF/human
	K. pneumoniae	8	CF/human
	K. pneumoniae	16	CF/human
	K. pneumoniae	16	CF/human
	K. pneumoniae	>=64	CF/human
	K. pneumoniae	>=64	CF/human
	K. pneumoniae	32	CF/human
	K. pneumoniae	4	CF/human
	K. pneumoniae	16	CF/human
	K. pneumoniae	4	CF/human
	K. pneumoniae	>64	CF/human
	K. pneumoniae	8	CF/human

	K. pneumoniae	16	CF/human
	K. pneumoniae	8	CF/human
	K. pneumoniae	4	CF/human
	K. pneumoniae	4	CF/human
	K. pneumoniae	16	CF/human
	K. pneumoniae	>64	CF/human
	K. pneumoniae	8	CF/human
	K. pneumoniae	8	CF/human
	K. pneumoniae	4	CF/human
	K. pneumoniae	8	CF/human
	K. pneumoniae	4	CF/human
	K. pneumoniae	32	CF/human
	K. pneumoniae	16	CF/human
	K. pneumoniae	32	CF/human
	K. pneumoniae	16	CF/human
	K. pneumoniae	16	CF/human
	K. pneumoniae	8	CF/human
	K. pneumoniae	8	CF/human
	K. pneumoniae	8	CF/human
	E. cloacae	16	KB/human
	E. cloacae	16	CF/human
	E. cloacae	16	CF/human
	E. cloacae	32	CF/human
	E. cloacae	>=64	CF/human
	E. cloacae	>=64	CF/human
	E. cloacae	16	CF/human
	E. cloacae	>=64	CF/human
	E. cloacae	>=64	CF/human
	E. cloacae	32	CF/human
	E. cloacae	>=64	CF/human
	E. cloacae	>=64	CF/human
	E. cloacae	8	CF/human
	E. cloacae	8	CF/human
	E. cloacae	>64	CF/human
	C. freundii	16	CF/human
	H. alvei	8	CF/human
	H. alvei	4	CF/human
	E. coli	8	A/animal
	E. coli	8	A/animal
	E. coli	16	A/animal
	E. coli	8	A/animal
	E. coli	8	A/animal
	E. coli	8	A/animal
	E. coli	8	A/animal
	E. coli	16	A/animal
	E. coli	16	A/animal
	E. coli	16	A/animal
	E. coli	8	A/animal
	E. coli	8	A/animal
	E. coli	16	A/animal
	E. coli	16	A/animal
	E. coli	8	A/animal
	E. coli	16	A/animal
	E. coli	8	A/animal
	E. coli	8	A/animal
	E. coli	16	A/animal
mcr-1 BLSE colistin susceptible	*E. coli*	<0.5	A/animal
mcr-1 BLSE colistin susceptible	*E. coli*	< 0.5	A/animal
mcr-3 BLSE colistin susceptible	*E. coli*	< 0.5	A/animal

mcr-4 BLSE colistin susceptible	*S. oneidensis*	1	KB/human
mcr-4 BLSE colistin susceptible	*S. profunda*	1	KB/human
non-MCR producing strains colistin susceptible	*E. coli*	0.25	KB/human
	E. coli	0.25	CF/human
	E. coli	0.25	CF/human
	E. coli	0.125	CF/human
	E. coli	0.25	CF/human
	E. coli	0.125	CF/human
	E. coli	0.125	CF/human
	E. coli	0.25	CF/human
	E. coli	0.25	CF/human
	E. coli	0.125	CF/human
	E. coli	0.5	CF/human
	E. coli	0.125	CF/human
	E. coli	0.125	CF/human
	E. coli	0.125	CF/human
	E. coli	0.25	CF/human
	E. coli	0.25	CF/human
	E. coli	0.25	CF/human
	E. coli	0.25	CF/human
	E. coli	0.5	CF/human
	E. coli	0.5	CF/human
	E. coli	0.5	CF/human
	E. coli	0.5	CF/human
	E. coli	0.5	CF/human
	E. coli	0.25	CF/human
	E. coli	0.25	CF/human
	E. coli	0.25	CF/human
	E. coli	0.25	CF/human
	E. coli	0.25	CF/human
	E. coli	0.25	CF/human
	E. coli	0.25	CF/human
	E. coli	0.25	CF/human
	E. coli	0.25	CF/human
	E. coli	0.25	CF/human
	E. coli	0.25	CF/human
	E. coli	0.125	CF/human
	E. coli	0.25	CF/human
	E. cloacae	0.5	CF/human
	E. coli	<0.5	A/animal
	E. coli	<0.5	A/animal
	E. coli	<0.5	A/animal
	E. coli	<0.5	A/animal
	E. coli	<0.5	A/animal
	E. coli	<0.5	A/animal
	E. coli	<0.5	A/animal
	E. coli	<0.5	A/animal
	E. coli	<0.5	A/animal
	E. coli	<0.5	A/animal
	E. coli	<0.5	A/animal
	E. coli	<0.5	A/animal
	E. coli	<0.5	A/animal
	E. coli	<0.5	A/animal
	E. coli	<0.5	A/animal
	E. coli	<0.5	A/animal
	E. coli	<0.5	A/animal
	E. coli	<0.5	A/animal
	E. coli	<0.5	A/animal
	E. coli	<0.5	A/animal

	E. coli	<0.5	A/animal
	E. coli	<0.5	A/animal
	E. coli	<0.5	A/animal
	E. coli	<0.5	A/animal
	E. coli	<0.5	A/animal
	E. coli	<0.5	A/animal
	E. coli	<0.5	A/animal
	E. coli	<0.5	A/animal
	E. coli	<0.5	A/animal
	E. coli	<0.5	A/animal

KB/human : human sample from Hôpital de Bicêtre; CF/human : human sample from CHU Clermont-Ferrand ; A/ animal : animal sample from ANSES Lyon
