# Supplementary material for: Development and Multicentric Validation of a Lateral Flow Immunoassay for Rapid Detection of MCR-1-Producing Enterobacteriaceae
Source: J Clin Microbiol. 2019 Apr 26;57(5):e01454-18. doi: 10.1128/JCM.01454-18 (PMC6498016; doi:10.1128/JCM.01454-18)
Supplement: Supplemental file 3 [file JCM.01454-18-s0003.pdf]

Supplemental Material and Methods 1: Production and characterization of anti-MCR-1 mAbs.

Ten-week-old Biozzi mice were immunized by intraperitoneal injection of purified recombinant p-MCR-1 (50 µg) as previously described (1). The immune response was followed by measurement of the anti-p-MCR-1 antibodies in sera using biotin-labeled p-MCR-1 as antigen. The two mice with the highest antibody titers were selected for mAb production. Spleen cells were fused with NS1 mouse myeloma cells (2), and specific anti-p-MCR-1 antibodies in myeloma culture supernatants were detected using the same immunoenzymatic test as the one previously used for the polyclonal response evaluation (3). After cloning of the cells according to Köhler and Milstein (4), mAbs were produced in and purified from culture supernatant by affinity chromatography using protein A and dialyzed in 0.05 M phosphate buffer pH 7.4.

Supplemental Material and Methods 2: Antibody selection for lateral flow format.

Preparation of colloidal gold-labeled MCR-1 antibodies. Colloidal gold was prepared as previously described (5) and 1 mL was centrifuged for 15 min at 15000 g and the pellet was suspended in water. 100 µL of a 100 µg/mL solution of each mAb (previously produced and selected, supplementary data) in 5 mM phosphate buffer pH 7.4 was added and incubated for 1 h at 20°C, allowing adsorption of the mAbs to the surface of the gold particles. Then, 100 µL of 20 mM phosphate buffer pH 7.4 and 1% BSA was added and the mixture was centrifuged for 15 min at 15000 g. The supernatant was discarded and the pellet resuspended in 1 mL of 2 mM phosphate buffer pH 7.4 and 0.1% BSA, sonicated for a few seconds and centrifuged for 15 min at 15000 g. The supernatant was discarded and the pellet was resuspended in 250 µL of 2 mM phosphate buffer pH 7.4, 0.1% BSA and stored at 4°C in the dark.

Strip screening test. To select the best mAb pairs for the development of the two-site lateral flow immunoassay, a combinatorial analysis was carried out using each mAb either as capture or gold-labeled antibody. Briefly, the strips were prepared by spotting 1 µL of mAb (100 µg/mL in 50 mM phosphate buffer pH 7.4) and then dried. 100 µL of a p-MCR-1 solution (50 ng/mL in EIA/0.1% Tween 20) or buffer alone and 10 µL of colloidal gold-labeled mAb were mixed in microtiter plate wells (Greiner) and allowed to react for 5 min before dipping the strip into the solution. After 30-min migration, signals were analyzed by the naked eye. The parameters used to select the best mAb pairs were the intensity of the visual signals obtained with a p-MCR-1 concentration (50 ng/mL) and the absence of signal without p-MCR-1 (non-specific signal).

In a second step, the same experiment was done with the best mAb pairs and MCR-1 extracted from *E. coli* strain. Briefly, an MCR-1-producing *E. coli* strain was grown in 1 L of LB until the $OD_{600\ nm}$ reached 1.6. The solution was centrifuged at 6000g for 20 min at 4°C and the pellet was suspended in 100 mL of extraction buffer (100 mM Tris-HCl pH 8, 0.15 M NaCl, 0.1% BSA, 0.5% Tween 20, 1% CHAPS). 100 µL of serial dilutions (1/10; 1/100 and 1/100) of the previously prepared solution in the extraction buffer or buffer alone and 10 µL of colloidal gold-labeled mAb were mixed in microtiter plate wells (Greiner) and allowed to react for 5 min before dipping the strip into the solution. After 30-min migration, signals were analyzed by the naked eye. The parameters used to select the best mAb pairs were the intensity of the visual signals obtained with the MCR-1 dilutions and the absence of signal without MCR-1.

Selection of best pairs. For this study we used a conventional strip format. The strips (0.5 cm in width and 4.5 cm in length) were composed of 3 parts: (i) a sample pad (Standard 14; Whatman) (0.5 cm in length), (ii) a nitrocellulose membrane (Prima 40) (2.5 cm in length), and (iii) an absorption pad (Cellulose grade 470; Whatman) (1.5 cm in length), all attached to a

backing card. The detection zone contained immobilized goat anti-mouse antibodies as a control line and an anti-p-MCR-1 mAb as a test line (1 mg/mL in 50 mM sodium phosphate buffer; pH 7.4) dispensed at 1 µL/cm using an automatic dispenser (Biojet XYZ 3050; BioDot, England). After drying for 1 h at 37°C in an air oven, the membrane was incubated with a blocking solution (PBS pH 7.4 containing 0.5% BSA) for 30 min at RT. The membrane was washed twice with deionized water, incubated for 20 min at RT in a preserving solution (PBS containing 0.1% Tween 20 and 7.5% glucose), and then dried for 20 min at 37°C in an air oven. After the absorption pad and the sample pad were fixed to the top and the bottom of the membrane, respectively, the card was cut into strips 5 mm in width using an automatic programmable cutter (CM4000 Guillotine cutting system; BioDot).

An MCR-1-producing *E. coli* strain was grown in 1 L of LB until the $OD_{600\ nm}$ reached 1.6. The solution was centrifuged at 6000g for 20 min at 4°C and the pellet was suspended in 100 mL of extraction buffer (100 mM Tris-HCl pH 8, 0.15 M NaCl, 0.1% BSA, 0.5% Tween 20, 1% CHAPS).

Each selected pair of antibodies was evaluated in the LFIA format using serial dilutions (1/10; 1/100 and 1/100) of the previously prepared solution in the extraction buffer. 100 µL of these dilutions was mixed for 5 min with 10 µL of the conjugate antibody before dipping the strip (for each pair of antibodies). The pair giving the highest signals for the highest dilutions was selected.

1. Simon S, Demeure C, Lamourette P, Filali S, Plaisance M, Créminon C, Volland H, Carniel E. 2013. Fast and Simple Detection of Yersinia pestis Applicable to Field Investigation of Plague Foci. PLoS ONE 8.

2. Grassi J, Frobert Y, Lamourette P, Lagoutte B. 1988. Screening of monoclonal antibodies using antigens labeled with acetylcholinesterase: Application to the peripheral proteins of photosystem 1. Anal Biochem 168:436–450.

3. Frobert Y, Grassi J. 1998. Screening of Monoclonal Antibodies Using Antigens Labeled with Acetylcholinesterase, p. 57–68. *In* Immunochemical Protocols. Humana Press, New Jersey.

4. Köhler G, Milstein C. 1975. Continuous cultures of fused cells secreting antibody of predefined specificity. Nature 256:495–497.

5. Khreich N, Lamourette P, Boutal H, Devilliers K, Créminon C, Volland H. 2008. Detection of Staphylococcus enterotoxin B using fluorescent immunoliposomes as label for immunochromatographic testing. Anal Biochem 377:182–188.
