# Supplementary figures and images for: Development and Multicentric Validation of a Lateral Flow Immunoassay for Rapid Detection of MCR-1-Producing Enterobacteriaceae
Source: J Clin Microbiol. 2019 Apr 26;57(5):e01454-18. doi: 10.1128/JCM.01454-18 (PMC6498016; doi:10.1128/JCM.01454-18)

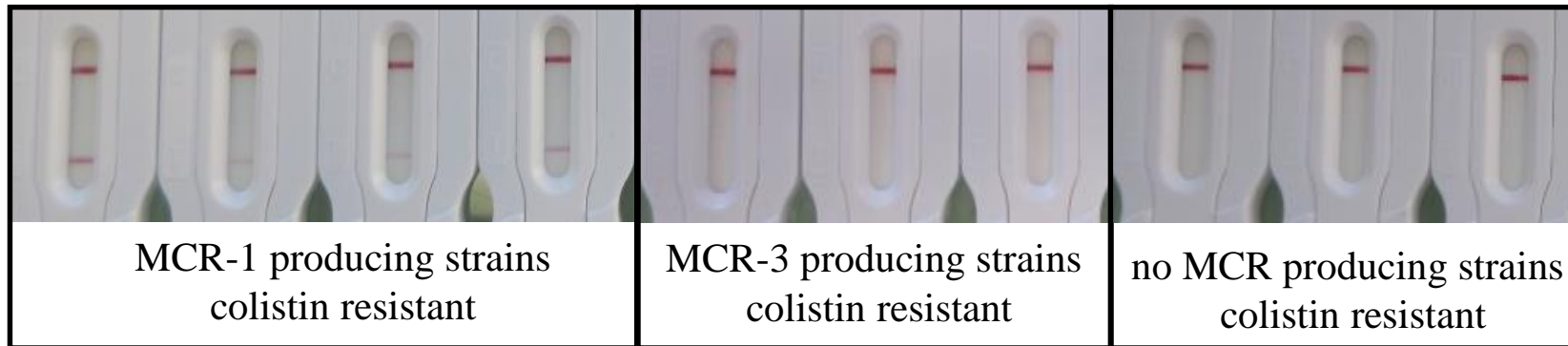

**Supplemental Figure 2:** Results obtained with different isolates

Supplement: Supplemental file 4 [file JCM.01454-18-s0004.pdf]
